# Transthyretin cardiac amyloidosis: advances and ambiguities

**DOI:** 10.1007/s10741-025-10552-9

**Published:** 2025-08-29

**Authors:** Alexandrina Danilov, Lorenzo D’Angelo, Enklajd Marsela, Juan Pablo Costabel, Ulrich P. Jorde, Yogita Rochlani

**Affiliations:** 1 Department of Medicine, Montefiore Medical Center, Albert Einstein College of Medicine, Bronx, NY USA; 2 Division of Cardiology, Montefiore Medical Center, Albert Einstein College of Medicine, Bronx, NY USA; 3 Instituto Cardiovascular de Buenos Aires, Buenos Aires, Argentina

**Keywords:** Cardiac amyloidosis, Heart failure, Cardiomyopathy, Artificial intelligence, Future directions

## Abstract

Cardiac amyloidosis is a fatal disorder caused by deposition of abnormally folded protein in the interstitial space. One of the proteins most associated with the disease is transthyretin (TTR), which leads to a progressive infiltrative cardiomyopathy (CM). Previously thought to be a rare disorder, there is growing recognition of it as a common cause of heart failure in the elderly and African Americans. The application of bone scintigraphy to the diagnosis of ATTR amyloidosis now allows for accurate and non-invasive diagnosis of the disease, rather than the previously necessary tissue biopsy. Targeted pharmacotherapies have been developed in the past few years that stabilize TTR, silence genes responsible for TTR production, or remove abnormal protein deposited in tissues. As of March 2025, Vutrisiran is the latest addition to the FDA-approved medications for ATTR-CM, alongside Tafamidis and Acoramidis. Several emerging therapies, including novel drugs and promising gene editing techniques are currently under investigation. As the number of available treatments continues to grow, maintaining a high index of suspicion and timely screening for the disease using laboratory tests, electrocardiography, and imaging has become increasingly important. In addition, with advancements in artificial intelligence (AI), new methods are in development to enhance screening of patients with suspected ATTR amyloidosis. These AI-driven tools could be integrated into electronic medical record systems to flag at-risk patients and allow for more rapid diagnosis. This review provides an overview of the current landscape and future directions of the diagnosis, treatment, and screening of ATTR-CM.

## Introduction

Amyloidosis is a heterogenous group of diseases characterized by misfolded protein deposition in the extracellular space, leading to multi-organ dysfunction. At least 35 precursor proteins have been implicated in causing amyloidosis, with misfolding resulting in formation of insoluble cross beta-sheets that aggregate into amyloid fibrils. Amyloidosis is categorized as localized or systemic depending on the organ(s) affected. Cardiac amyloidosis is one of the most devastating systemic amyloidoses, causing an infiltrative, restrictive cardiomyopathy and eventually heart failure (HF) often co-existing with arrhythmias and conduction abnormalities. Two main types of amyloid cardiomyopathy (CM) exist: amyloid light chain (AL), resulting from excess light chain production from plasma cell dyscrasias, and amyloid transthyretin (ATTR), resulting from abnormally folded TTR protein produced by the liver. ATTR-CM is further subdivided into variant or hereditary (ATTRv), previously known as familial, and wild-type (ATTRwt), previously known as senile [1].

**Fig. 1:**
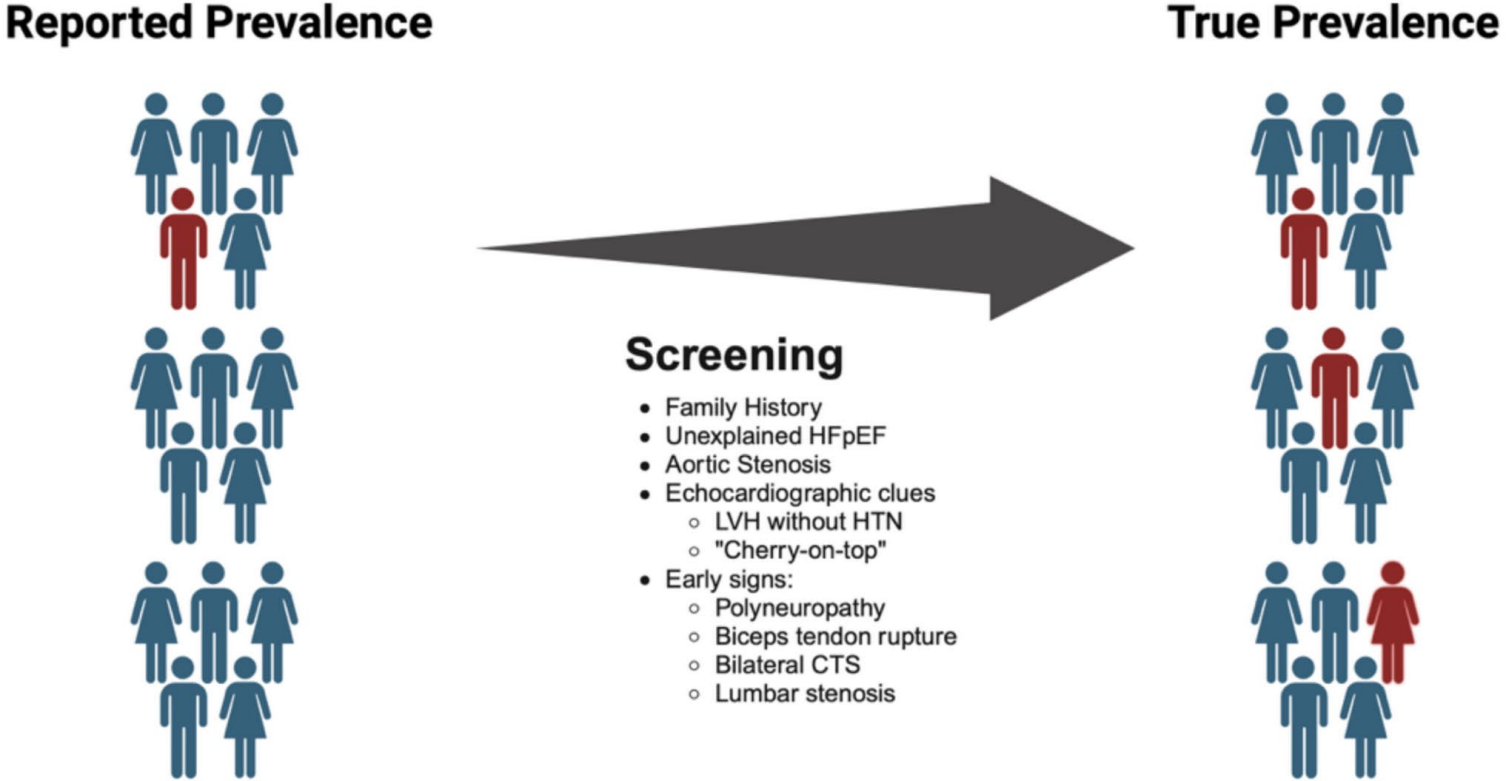
The true prevalence of ATTR is likely higher after screening for certain at-risk populations (HFpEF = heart failure with preserved ejection fraction; LV = left ventricle; HTN = hypertension; CTS = carpal tunnel syndrome)

Classically considered rare, recent advancements in screening and diagnosis have allowed greater recognition of the disease and uncovered a prevalence higher than previously thought, Fig. 1 [2]. The exact prevalence is unknown; however, among Medicare beneficiaries in the United States (US), which comprises a majority of the US population ≥ 65 years, hospitalized with HF, the prevalence increased from 8 to 17 per 100,000 person-years from 2000 to 2012 [2]. Based on the conservative assumption that 4% of adults above the age of 60 with heart failure with preserved ejection fraction (HFpEF) have ATTR-CM, the prevalence is 120,000 in the US [3]. Additionally, studies in patients with comorbidities such as aortic stenosis undergoing percutaneous valve replacement procedure report a prevalence of 15% [4].

**Fig. 2:**
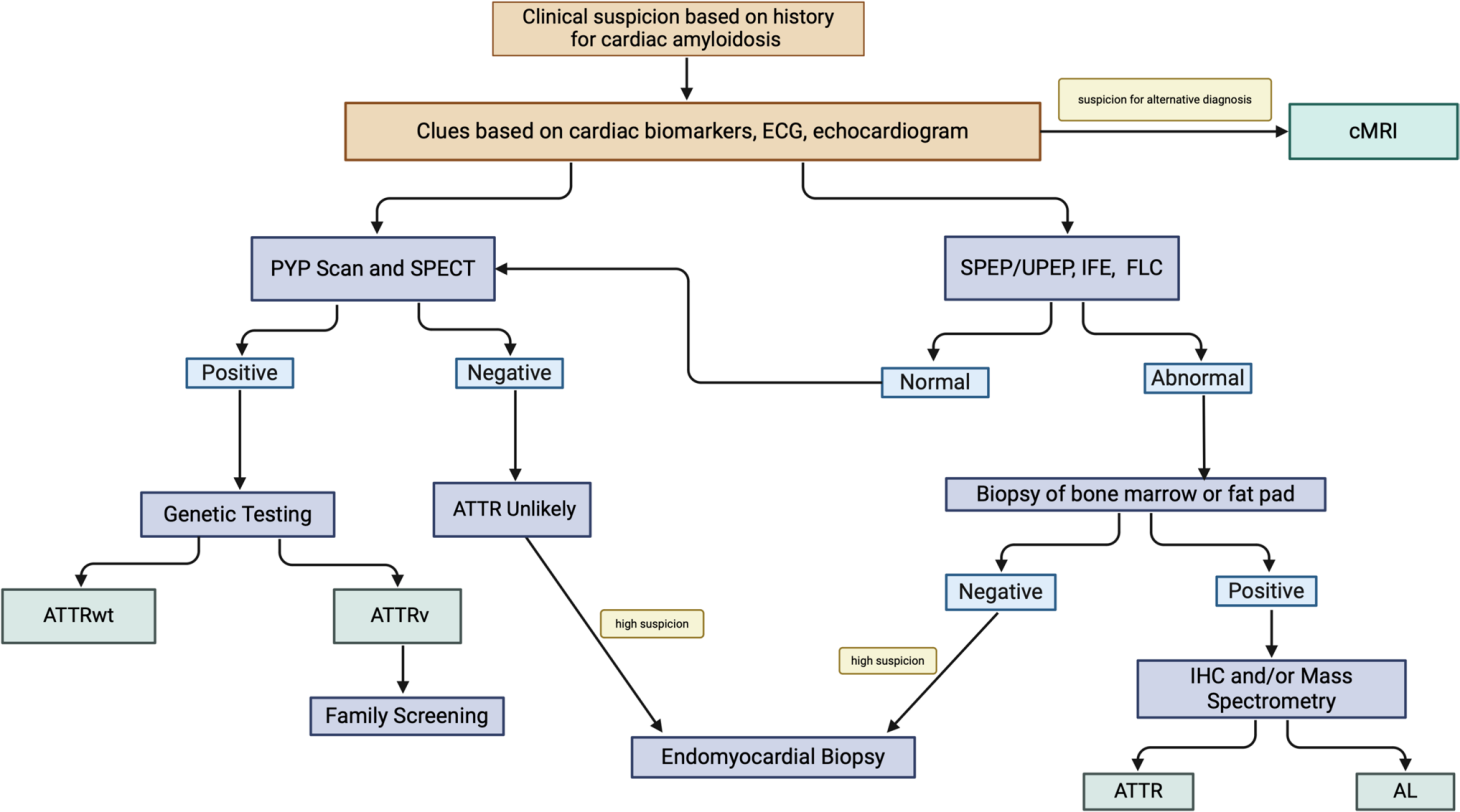
Diagnostic pathway of amyloidosis (ECG = electrocardiogram; MRI = magnetic resonance imaging; SPEP = serum protein electrophoresis; UPEP = urine protein electrophoresis; IFE = immunofixation; FLC = free light chains; PYP = pyrophosphate; SPECT = single photon emission computed tomography; ATTR = amyloid transthyretin; AL = amyloid light chain; IHC = immunohistochemistry)

Transthyretin, also called prealbumin, is a thyroxine and retinol transport protein produced by the liver, as well as to a lesser extent by the choroid plexus and retinal pigmented epithelium. It naturally exists in tetramer form, but its pathologic dissociation into monomers due to aging or genetic mutations leads to amyloid fibril formation, Fig. 2. The rate of misfolded wild-type TTR increases with age, leading to ATTRwt, the overall most common form of ATTR occurring in 25% of people above the age of 80 based on anatomopathological studies [5, 6]. On the other hand, various autosomal dominant single-nucleotide polymorphisms in the *TTR* gene cause ATTRv. The most common mutation in the US is a substitution of isoleucine for valine at position 122 of the TTR protein sequence (Val122Ile). A polymorphism unique to those of African descent, its prevalence is roughly 3.5% in the African American population [7, 8]. Mutations differ across populations: Val30Met and Thr60Ala are other commonly studied polymorphisms present in up to 2% of Swedes and ~ 1% of those of Irish descent, respectively [9, 10].

Despite autosomal dominance, variable genetic penetrance makes it difficult to predict who will develop the disease and when. Clinical penetrance depends on age, sex, and allele variant [11]. The penetrance of the Val122Ile variant has not been firmly established, but generally increases after 65 years of age with a lower penetrance observed in women [12]. Anatomic penetrance has been observed to be as high as ~ 80% in African American men based on echocardiographic and electrocardiographic changes [13]. More recent data from the SCAN-MP study showed phenotypic penetrance to be 39% in self-identified Black and Hispanic individuals at or above 60 years of age with heart failure and increased left ventricular (LV) wall thickness [14]. In contrast, another study observed a much lower clinical penetrance and no significant sex difference, despite more subclinical abnormalities in allele carriers [15]. The hereditary Val30Met variant ranges in penetrance from ~ 15% at the age of 50 in Swedish and French individuals to ~ 80% at the age of 50 in Portuguese individuals [9, 16].

Additionally, ATTR amyloidosis can clinically manifest with neuropathy or cardiomyopathy in the form of HFpEF or both, which are common comorbidities seen in older adults and can result from several other etiologies making it difficult to diagnose and leading to delays or misdiagnoses. Both ATTRv and ATTRwt affect men disproportionately more than women, but the average age at diagnosis is approximately 51 and 75 years, respectively, potentially indicative of the more indolent disease progression in ATTRwt. Median survival after diagnosis in untreated ATTR-CM is up to 2.8 years in those with the Val122Ile variant, 4.8 years in wild-type, and 5.8 in other variants, although survival is dependent on the degree of cardiac involvement [17]. Historically, diagnosis of the disease required tissue biopsy and treatment was limited, focusing on liver transplant to remove the source of the abnormal protein and/or heart transplant. The rise of non-invasive diagnostic and screening methods in addition to growing interest in targeted pharmacotherapies has led to major progress in ATTR-CM in recent years [18–22].

## Diagnosis

Cardiac amyloidosis should be suspected in patients presenting with unexplained HFpEF, particularly in the presence of discordance between moderate to severe LV wall thickening (≥ 14 mm) and unexpectedly low QRS voltages. Additional red flags include persistent low-level elevation in serum troponin, unexplained AV-block, or prior pacemaker implantation. History of carpal tunnel syndrome (particularly if bilateral in a male), atraumatic rupture of biceps tendon, autonomic dysfunction, or unexplained neuropathy may also be systemic clues. In elderly patients, particularly those of African American descent, the presence of these findings should raise suspicion for ATTR-CM, while younger individuals with multisystem involvement warrant evaluation for AL amyloidosis [23, 24].

Definitive diagnosis was historically made with histochemical staining of affected tissue using the Congo red stain, which marks beta-folded sheets and exhibits apple-green birefringence under polarized light. Immunohistochemistry or tandem mass spectrometry then determines exactly which type of protein is involved: AL or ATTR [25]. Tissue can be obtained from various organs, given the multisystem involvement, such as the heart and abdominal fat pad, among others; however, the gold standard for diagnosis of cardiac amyloidosis remains the endomyocardial biopsy. In the past few decades, major advancements have been made in the diagnosis of ATTR-CM using multimodal imaging, which in many cases has allowed clinicians to dispense with the need for invasive biopsy, which can be hard to access outside of large or academic centers and is associated with procedural risk due to its invasive nature.

Once there is suspicion for cardiac amyloidosis due to history, electrocardiographic, biochemical, or echocardiographic “red flags,” protein electrophoresis with immunofixation of the serum and urine as well as serum free light chains should be measured. If elevated, AL amyloid is more likely, and biopsy of the abdominal fat pad or other accessible tissue such as heart or bone marrow should be performed for histologic diagnosis. If no monoclonal protein is detected, nuclear imaging using ^99m^Tc-pyrophosphate (PYP), a radiotracer that localizes to the bones and structures accumulating ATTR, can clarify the diagnosis. Interpretation is based on comparison of cardiac uptake to that of the ribs using the semiquantitative visual score: Grade 0 (no cardiac uptake), Grade 1 (cardiac less than rib uptake), Grade 2 (cardiac equal to rib uptake), or Grade 3 (cardiac greater than rib uptake). Alternatively, a quantitative score of the heart-to-contralateral chest (H/CL) ratio may be used.

A diagnosis of ATTR-CM is considered if there is Grade 2 or 3 uptake, or the H/CL ratio is ≥ 1.5 at one hour of incubation or ≥ 1.3 after 3 h of incubation [26]. Single photon emission computed tomography (SPECT) imaging is preferred over planar imaging to rule out false positives due to blood pooling in the LV and confounding by overlying chest tissue. PYP scans have high specificity and sensitivity in multicenter studies (at least 91% and 92%, respectively) for ATTR and can differentiate ATTR from non-amyloid heart disease [26, 27]. In a population with high prevalence, the negative predictive value (NPV) was 81% and positive predictive value (PPV) was 96% [26]. In another cohort, the combination of a positive PYP scan (Grade 2 or 3 uptake) and lack of a monoclonal protein had a specificity and PPV for ATTR-CM up to 100%, emphasizing the importance of measuring monoclonal protein levels prior to the scan [19]. Bone scintigraphy provides no information about the structure and function of the heart; cardiac magnetic resonance imaging (cMRI) should be performed if there is suspicion for an alternative diagnosis. The exact mechanism by which the PYP scan identifies ATTR is not well understood and there have been reports of false positive studies in cases with LV hypertrophy associated with Plaquenil cardiomyopathy or hypertrophic cardiomyopathy [28, 29]. Of note, ^99m^Tc-hydroxymethylene diphosphonate (HMDP or HDP) can be used as an alternative bone-avid radiotracer in clinical practice when PYP is unavailable [30].

Molecular imaging is currently an area of active investigation with the goal of enabling earlier and more precise diagnosis. Several ^18^F-labeled amyloid-specific positron emission tomography (PET) radiotracers such as florbetapir, flutemetamol, and florbetaben which bind to beta-pleated amyloid sheets were originally used for Alzheimer’s disease and are now increasingly being studied in cardiac amyloidosis [31]. Peptides that binds to heparan sulfate proteoglycans within amyloid fibrils, ^123^I-evuzamitide (SPECT-based) and ^124^I-evuzamitide (PET-based) are also actively being investigated [32]. However, these radiotracers have not been shown to distinguish between AL and ATTR amyloid, although it has been suggested that ^124^I-evuzamitide detects ATTRwt better than ^18^F-florbetapir [33].

Endomyocardial biopsy can now be avoided in most cases if bone scintigraphy is available and diagnosis is clear based on bloodwork and noninvasive imaging; however, if noninvasive testing is unavailable or PYP scan is negative despite a high clinical suspicion, biopsy should be considered. Once diagnosis is established, genotyping is crucial to establish the expected course and best treatment, as certain mutations are known to be more aggressive, such as Val122Ile [34]. If the patient is found to have ATTRv, genetic testing should be offered to first degree relatives, Fig. 2.

## Treatment

### Role of guideline-directed medical therapy (GDMT)

Prior to the advent of disease modifying therapies, treatment of ATTR-CM was limited to alleviating the symptoms of congestive HF and arrhythmias, generally using short-term temporizing measures. Aldosterone antagonists and loop diuretics are often used to maintain euvolemia. In observational studies, sodium-glucose co-transporter-2 inhibitors were shown to improve mortality and decrease hospitalizations [35]. However, beta blockers and calcium channel blockers are poorly tolerated as the decline in heart rate with a fixed stroke volume in restrictive CM could lead to a decline in cardiac output. Angiotensin converting enzyme inhibitors or angiotensin receptor blockers are usually not well tolerated due to orthostatic hypotension resulting from autonomic dysfunction and do not have any established benefit in ATTR-CM.

### Arrhythmias and conduction abnormalities

Amyloid CM is also highly associated with atrial and ventricular arrhythmias, with the most common being atrial fibrillation. Patients generally require ambulatory rhythm monitoring. Anticoagulation for atrial fibrillation and flutter is indicated in patients regardless of CHADS-VASC score given inherent atrial dysfunction. Amiodarone is preferred for both rhythm and rate control, especially in patients who cannot tolerate beta blockers [36]. While there have been no major trials investigating digoxin in amyloid CM and older studies express concern regarding a lower threshold for digoxin related toxicity due to amyloid fibril binding, a more contemporary retrospective analysis shows that it could be used in select patients for rate control with arrhythmias [37]. Direct current cardioversion and ablation can also be considered. Implantable cardioverter-defibrillators may be considered for prevention of sudden death from ventricular arrhythmias, although no studies have shown benefit for either primary or secondary prevention [36]. Amyloid infiltration of the conduction system often leads to conduction abnormalities, and cardiac resynchronization therapy with defibrillator may be helpful in pacemaker-dependent patients [38].

### Devices and advanced therapies

**Fig. 3:**
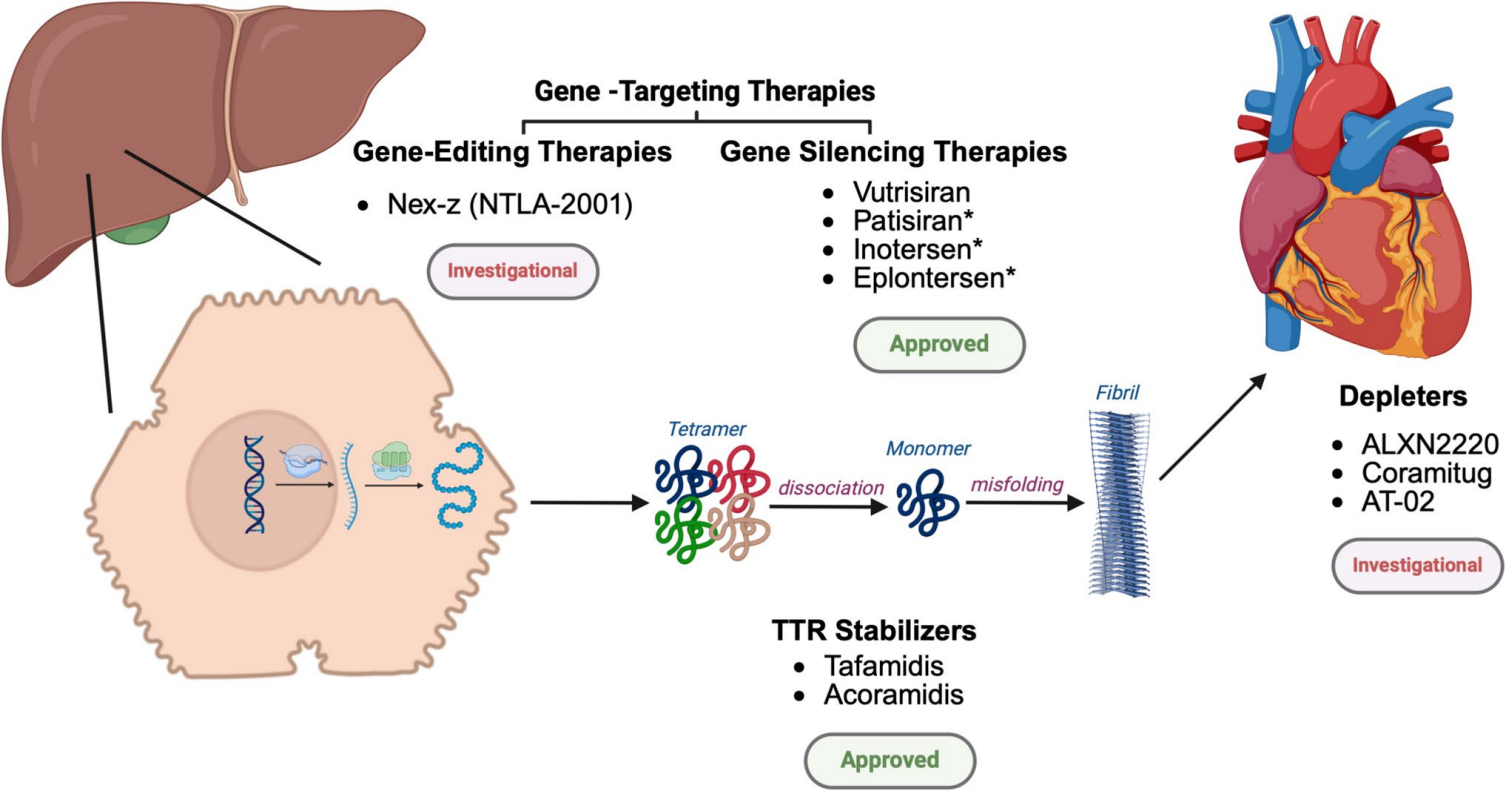
FDA-approved and investigational therapies for transthyretin amyloidosis. *Currently approved only for polyneuropathy of hereditary transthyretin amyloidosis

Options for advanced mechanical circulatory support devices in end-stage HF due to amyloid are minimal given the small size of the LV and biventricular dysfunction. Nonetheless, novel ventricular assist device techniques involving transseptal left atrial cannulation via the right atrium show promise as an alternative that effectively unloads the LV in restrictive CM [39]. The definitive treatment for end stage HF due to amyloid is heart transplant, which has increased survival of the disease by over 10 years, and was historically sometimes performed alongside liver transplant for ATTR-CM to eliminate the source of abnormal protein production [40]. The heart transplant allocation system, managed by the United Network for Organ Sharing (UNOS), prioritizes patients based on medical necessity using a 6-tier system, with Status 1 being the most urgent. Given the limited options for mechanical circulatory support and the high mortality rate observed while on the waiting list, patients with ATTR-CM are now given preference at Status 4 [41]. However, organ transplant comes with myriad restrictions, such as organ availability and transplant-associated complications, providing the opportunity for new approaches. Pharmacological therapies have recently come to the forefront of amyloid treatment. The three mainstays of molecular therapies in ATTR-CM are ATTR stabilizers, silencers, and eliminators or depleters, Fig. 3.

### Disease-modifying therapies

Treatments targeting the molecular pathophysiology of ATTR-CM have changed the trajectory of the disease and raised more questions regarding their use throughout the disease course. These therapies have been extensively discussed previously [42, 43]. Here, we provide a brief summary and highlight how recently emerging data influences clinical decision-making. Stabilizers function by stabilizing the TTR tetramer and inhibiting its dissociation into monomers. Tafamidis, approved by the FDA in 2019, and Acoramidis, approved in 2024, have both been shown to reduce mortality, cardiovascular-related hospitalizations, functional decline, and cardiac dysfunction [20, 21]. The ATTR-ACT trial investigating Tafamidis included 441 predominantly male patients with majority wild-type ATTR-CM at an average age of 75 years. Patients with New York Heart Association (NYHA) Class IV symptoms were excluded. The Tafamidis group had lower all-cause mortality with a hazard ratio of 0.70 (95% confidence interval (CI): 0.51–0.96) and cardiovascular-related hospitalizations with a relative risk of 0.68 (95% CI: 0.56–0.81). However, when analyzing subgroups, NYHA Class III had more cardiovascular-related hospitalizations in the Tafamidis group, speculated to be secondary to longer survival during advanced disease. Differences in secondary end points of distance walked in the 6-min walk test and the Kansas City Cardiomyopathy Questionnaire overall summary (KCCQ-OS) score were seen starting at 6 months and mortality benefit was seen at 18 months [20]. Persistent mortality benefit was demonstrated in the long-term extension study at a median follow-up of ~ 58 months [44].

The ATTRibute-CM trial, comparing Acoramidis to placebo, included 632 patients with similar baseline characteristics to ATTR-ACT and favored Acoramidis in a four-step primary hierarchical analysis based on all-cause mortality, cardiovascular-related hospitalizations, change in N-terminal pro–B-type natriuretic peptide (NT-proBNP), and change in 6-min walk distance with a win ratio of 1.8 (95% CI: 1.4–2.2) at a follow up of 30 months. Mortality benefit with Acoramidis was seen starting at approximately 19 months, similarly to the tafamidis trial [21]. However, most participants of both trials had ATTRwt, potentially limiting its generalizability to other forms of cardiac amyloid, although a follow-up analysis of the ATTR-ACT trial showed no difference in mortality benefit between ATTRv and ATTRwt [45]. In a post-hoc analysis, Tafamidis was also found to lessen the decline of several echocardiographic metrics of cardiac function: LV stroke volume, global longitudinal strain, septal E/e’ ratio, and lateral E/e’ ratio, but not LV ejection fraction [46].

Several other TTR stabilizers have been explored. Diflunisal is a non-steroidal anti-inflammatory agent used off-label for the treatment of ATTR amyloidosis after it was found to stabilize TTR in vitro. Although no large-scale randomized controlled trials have been conducted in ATTR-CM, it has been associated with decreased mortality and transplant rates [47]. Tolcapone, approved for the treatment of Parkinson’s disease, also stabilizes the TTR tetramer, although its use is limited by a multitude of side effects [48].

Gene-silencing therapies, via either RNA interfering agents or antisense oligonucleotides that stop production of the deleterious protein have been studied in the treatment of ATTR amyloid, such as Patisiran, Vutrisiran, Inotersen, and Eplontersen [18, 22, 49, 50]. These agents have predominantly been evaluated for ATTRv polyneuropathy and two of these drugs have also been studied in amyloid CM. Patisiran showed improvement in functional capacity and LV wall thickness, but not in mortality or cardiovascular events [22]. Vutrisiran was recently studied in the HELIOS-B trial and included 655 patients with ATTR-CM, the majority being men with ATTRwt in NYHA Class II [18]. Benefit in recurrent cardiovascular events and all-cause mortality started at approximately 6 months with Vutrisiran therapy, and there was a slower decline in functional capacity via the 6-min walk test and KCCQ-OS score. Given these positive results, Vutrisiran was approved by the FDA for use in ATTR-CM in March of 2025 [18]. There are no head-to-head trials comparing stabilizers with silencers yet, and in fact 40% of the patients enrolled in the HELIOS-B trial were on background Tafamidis therapy. Results from the study show similar outcomes in those on Vutrisiran monotherapy compared to those on Vutrisiran plus Tafamidis [18].

Cardiac TTR amyloid depleters, which clear amyloid fibrils, form a third major class of new drugs that show promise as disease-modifying therapy in amyloidosis. Traditionally, this class included doxycycline plus tauroursodeoxycholic acid, which increases clearance of misfolded TTR deposits but has not been effective in ATTR-CM due to side effects and minimal efficacy, and epigallocatechin-3-gallate, a substance found in green tea which increases clearance in vitro but has not led to significant differences in humans [51, 52]. The monoclonal antibody ALXN2220, formerly known as NI006, a recombinant human anti-ATTR antibody which removes ATTR from cardiac tissue using phagocytic immune cells, is currently undergoing testing in Phase 3 trials as part of the DepleTTR-CM Study after a Phase 1 trial established its safety [53]. After 12 months, there was apparent decrease of cardiac tracer uptake via scintigraphy and extracellular volume on cMRI, as well as pro-BNP and troponin T [53]. Another monoclonal antibody coramitug, previously called NNC6019 and PRX004, which also functions by increasing TTR clearance via phagocytosis, is currently being studied. A Phase 2 trial investigating coramitug to establish the optimal dosing was recently completed and a Phase 3 trial is planned to commence by the end of the 2025 [54]. PRX004 is a humanized monoclonal antibody previously undergoing investigation; however, a Phase 1 trial was terminated early due to the Covid-19 pandemic [55].

Lastly, a more novel approach to pharmacotherapy uses the clustered regularly interspaced short palindromic repeats (CRISPR) and CRISPR-associated protein 9 (Cas9) gene editing system to reduce the level of TTR protein produced by the liver. This technique uses a guide RNA to direct the Cas9 endonuclease enzyme to a DNA sequence and then cut or replace the gene of interest [56]. The NTLA-2001 system, now known as nexiguran ziclumeran (nex-z), which uses this novel technology, was shown to be safe and lowered serum TTR levels in a small group of participants with ATTRv with polyneuropathy, with or without cardiac involvement [57]. In another Phase 1 trial in 36 individuals with ATTR-CM who received a single intravenous infusion of nex-z, there was −90% change in ATTR serum levels at 12 months and no significant change in cardiac biomarkers, but significant infusion-related reactions occurred [58]. There is currently a Phase III trial underway to evaluate the effect of this system on mortality, cardiovascular events, serum TTR, and functional status in participants with ATTR-CM [59].

Disease-modifying therapies must be initiated as early as possible in the disease course to elicit benefit in mortality and quality of life. Both Tafamidis and Acoramidis are limited to patients in NYHA Classes I-III, as Class IV individuals were excluded from initial trials and the drugs are expected to slow down disease progression rather than reverse it [20, 21]. Disease at this late stage is likely far too advanced with irreversible myocardial damage, such that prevention of further amyloid deposition would yield minimal results. Furthermore, the HELIOS-B trial included NYHA Classes I-III, but most individuals at baseline were in NYHA Class II and most benefit with Vutrisiran was seen for those in NYHA Class I or II [18]. While recent trials have clearly shown improved outcomes, personalized care that reflects disease stage, genotype, and goals of care must be emphasized.

## Screening

Early screening, including family members of affected individuals with ATTRv, and diagnosis of cardiac amyloid is essential, given that disease can be potentially prevented or significantly slowed through early stabilizer, gene-silencing, gene-editing, or antibody-based therapy. The transthoracic echocardiogram is often the first step in evaluation and is characterized by globally increased wall thickness, decreased chamber dimensions, and diastolic and/or systolic dysfunction. LV hypertrophy defined as wall thickness ≥ 14 mm on echocardiogram should raise alarm in patients without hypertension and prompt evaluation for a broad differential, including cardiac amyloidosis. Prior to any reduction in ejection fraction, which is often seen late in the disease course, speckle-tracking echocardiography can visualize impaired longitudinal strain. A distinguishing feature of cardiac amyloidosis is abnormal longitudinal strain with apical sparing relative to the basal and mid segments of the LV, a “cherry-on-top” pattern [60].

LV hypertrophy is an immediately recognized but non-specific feature of ATTR-CM shared by several other conditions including hypertrophic cardiomyopathy, Fabry’s, or athlete’s heart. Given this overlap, several other echocardiographic “red flags” specific to amyloid CM have been investigated. In the AC-TIVE study, the prevalence of amyloid CM in a group of individuals ≥ 55 years old with left ventricular hypertrophy and HFpEF and one additional red flag (including interatrial septum thickness, pericardial effusion, restrictive filling pattern, granular sparkling, atrio-ventricular valve thickness, or apical sparing) was 29%, with the majority being ATTR. Apical sparing or at least two echocardiographic red flags (excluding interatrial septal thickness) resulted in a high diagnostic accuracy > 70%, which was higher when including the clinical scenario [61]. While echocardiography contributes to suggesting the diagnosis, it lacks specificity and sensitivity.

If there is suspicion for an alternative diagnosis, cMRI can help differentiate amyloid CM from other hypertrophic processes. The classic cMRI pattern in cardiac amyloidosis is the diffuse or, less commonly, patchy late gadolinium enhancement throughout the myocardium, which can provide significant prognostic value [62, 63]. However, it is difficult to quantify, a problem which T1 mapping, a technique that measures tissue relaxation time, bypasses. Native T1 mapping (pre-gadolinium contrast) demonstrates areas of amyloid deposition and can identify early disease in patients without late gadolinium enhancement [64]. Post-contrast T1 mapping after gadolinium administration can estimate the extracellular volume, which is elevated in amyloid CM, but not specific to the disease [65]. Another important cMRI feature that identifies amyloid CM with high sensitivity is nulling of the myocardium prior to the blood pool on the T1 scout series, which is suggestive of global hyperenhancement [66].

Certain risk assessment scores have been created to determine who should undergo diagnostic workup using nuclear bone scintigraphy. Davies et al. developed and validated the ATTR-CM score using clinical (age, male sex, hypertension) and echocardiographic (ejection fraction, posterior wall thickness, relative wall thickness) variables to predict risk of ATTR-CM in HFpEF (ejection fraction ≥ 40%) cohorts with variable prevalence. The score had strong discrimination and calibration, with a score of ≥ 6 being clinically significant with a PPV of ≥ 25% in a population with a HFpEF prevalence of ≥ 10% [67]. Bodrini et al. developed a multiparametric echocardiography risk score (including relative wall thickness, E wave/e′ wave ratio, longitudinal strain, tricuspid annular plane systolic excursion, septal apical–to–base ratio) that accurately predicted cardiac amyloidosis (both AL and ATTR) in patients with increased wall thickness [68]. The T-Amylo prediction model—using age, gender, carpal tunnel syndrome, interventricular septal thickness in diastole, and low QRS voltages—was the basis for a simplified score that performed well in diagnosing ATTR-CM in cohorts with hypertensive cardiomyopathy, severe aortic stenosis, and HFpEF [69]. Risk prediction scores are important tools in the initial screening of patients with suspected ATTR-CM and have the potential to become standard practice after further validation in larger cohorts.

## Use of artificial intelligence in detecting cardiac amyloidosis

Screening for cardiac amyloidosis includes new advancements in artificial intelligence (AI) that use data to predict diagnosis or treatment. AI is the ability of machines to perform tasks that typically require human intelligence, such as learning, reasoning, and predicting. Its use has been increasingly studied in medicine. Machine learning (ML) is a type of AI that trains models to study data and make predictions without explicit programming by humans. Deep learning (DL) is a subtype of ML that uses artificial neural networks to process data and make predictions. Considering the difficulties and delays in diagnosing amyloidosis, the growth of AI could be pivotal, and many models have already been created.

Recently, Grogan et al. trained a deep neural network model to predict cardiac amyloidosis based on ECG data. The model accurately diagnosed 84% of patients with amyloidosis, which predated clinical diagnosis by 6 months in 59% of cases [70]. Echocardiographic changes in amyloidosis can be subtle to the naked eye, have inter-operator variability, and are similar to those in other disease processes, providing another avenue for AI assistance. ML models have been created that accurately detect diseases such as cardiac amyloidosis by estimating LV mass and mitral annulus e’ velocity [71]. One study used both ECG and echocardiogram data to create a model that accurately predicted the presence of cardiac amyloidosis. The model was better at diagnosing ATTR than ALL; could differentiate cardiac amyloid from hypertension, end-stage renal disease, and hypertrophic cardiomyopathy; and outperformed human cardiologists [72]. Other models have been created that predict cardiac amyloisis using cMRI, nuclear imaging, and histology [73–76]^.^ In addition to facilitating workflow and allowing for higher patient volumes, ML models have the potential to accurately diagnose and predict disease when compared to human clinicians. Further studies are needed to validate these tools in clinical practice and ensure their generalizability to the general population.

## Challenges and future directions

Despite the advances in screening, diagnostic methods, and available treatment options for patients with ATTR-CM, several challenges remain. When discussing screening methods, we mentioned that AI-based screening tools have been proposed to diagnose ATTR-CM in its earliest possible stages. However, though promising, they come with unique difficulties for their application and interpretation. One of the main challenges in applying these tools is that current studies are predominantly retrospective, single-center, and based on limited, homogeneous datasets, raising concerns about their generalizability [77]. For instance, the deep neural network model developed at the Mayo Clinic to predict cardiac amyloidosis based on ECG data was found to have a high NPV (> 90%) in a validation study; however, the low prevalence of amyloidosis resulted in lower PPV in the general population [78]. This shows that real-world application of these screening tools is limited and that they might work better in a high prevalence population with a high pre-test probability. In addition, applying them in a non-selected population can result in a large amount of false positive results and might lead to confusion in further management as well as burden non-amyloid specialists.

Another significant barrier is the"black box"nature of deep learning algorithms, limiting clinician trust due to their lack of transparency in decision-making processes. Practical considerations, such as workflow integration, standardization across platforms, and regulatory approval, also pose challenges [77]. There is promising evidence that AI tools can greatly assist in the screening process and lead to early and accurate diagnosis; however, prospective studies are needed to demonstrate improved patient outcomes, validate performance across diverse populations, and ensure smooth integration into healthcare practices.

With growing awareness and improved screening, clinicians are increasingly identifying ATTR-CM at earlier stages, raising the question of when to initiate treatment in asymptomatic patients [79]. Early identification offers a chance to intervene before irreversible cardiac damage occurs; however, a major evidence gap remains. The challenge encountered here is that to date, pivotal trials have only included symptomatic patients, leaving uncertainty around the benefits of early ATTR-CM treatment [18, 20]. Current practices typically involve close monitoring and shared decision making, leaning towards treatment initiation upon symptom onset or biomarker changes. This may be partly due to lack of evidence, high therapy costs, and uncertain long-term benefits of starting treatment very early. As a result, management of asymptomatic ATTR-CM remains inconsistent. For now, the importance of early detection is clear (since it prompts vigilance and timely therapy at symptom onset), but the optimal timing of therapy initiation in asymptomatic ATTR-CM remains an open question and a priority for future research [79, 80].

After establishing the diagnosis, and once the decision to treat ATTR-CM is made, providers might encounter additional challenges. With the rapidly evolving clinical landscape of ATTR-CM, and as more treatment options emerge, choosing an initial disease modifying therapy (or therapies) has become clinically complex. Clinicians currently navigate multiple therapeutic options and must decide, for example, whether to initiate treatment with stabilizers or silencers alone or in combination or other investigational therapies that may soon be approved. However, there is no head-to-head trial guiding initial therapeutic choices, emphasizing individualized treatment decisions based on patient comorbidities, preferences for drug delivery, drug profiles, and cost considerations.

Furthermore, as more therapies emerge for ATTR-CM, the idea of combination therapy—targeting multiple points in the pathogenic cascade simultaneously—is drawing attention [81]. In theory, a multi-pronged approach could yield additive benefits by attacking the disease on multiple fronts. This leaves uncertainties around the benefits, long-term safety, adherence, and cost-effectiveness of combination therapy [82].

After treatment is initiated, monitoring is based on clinical changes, biomarkers, or cardiac imaging; to assess therapeutic response. However, we lack a method of directly quantifying effects of treatment on misfolded TTR prior to manifestation of downstream effects. Development of a surrogate marker of treatment efficacy would guide therapeutic decision-making and further individualize treatment strategies.

Finally, despite breakthroughs in ATTR-CM therapy, the extraordinary cost of systematic screening and medications significantly adds to the challenges of equitable care delivery [82, 83]. Addressing affordability through policy changes and broader insurance coverage is essential.

In this dynamic context, managing patients with ATTR-CM will require a personalized and multidisciplinary approach [84], particularly as workup is often initiated by specialists including hematologists, orthopedic surgeons, neurologists, and geriatricians [85]. Effective care requires actively engaging patients in decisions, clearly communicating treatment trade-offs, and aligning therapy with each patient’s values and treatment goals. Future efforts should focus on generating comparative clinical evidence, developing practical clinical decision pathways, and promoting shared decision-making.

## Conclusion

Cardiac amyloidosis is a progressive and deadly disease that has garnered increasing interest in recent years. The development of non-invasive diagnostic methods, recognition of a higher prevalence than previously thought, and novel targeted pharmacotherapies from TTR stabilizers to gene editing has led to greater recognition of the disease along with improved outcomes. Certain populations are increasingly recognized as at risk for the disease. Considering the importance of initiating early treatment, the development of accurate and high-throughput screening tools will be of consequence. New screening methods, including AI-based, have the potential to change future evaluation and management of the disease. Further research comparing therapeutic options that can guide clinicians in selecting the best treatments for their patients and regulatory changes to lower the financial burden of these medications is crucial.

## Data Availability

No datasets were generated or analysed during the current study.
